# Supplementary material for: Hybrid Models and Biological Model Reduction with PyDSTool
Source: PLoS Comput Biol. 2012 Aug 9;8(8):e1002628. doi: 10.1371/journal.pcbi.1002628 (PMC3415397; doi:10.1371/journal.pcbi.1002628)
Supplement: Text S4 — Complete source code for the PyDSTool package (version 0.88.120504). Includes API documentation and help files linking to web pages. This file is identical to the current public release on Sourceforge.net. (ZIP) [file pcbi.1002628.s004.zip › PyDSTool/html/PyDSTool.Toolbox.makeSloppyModel-pysrc.html]

xml version="1.0" encoding="ascii"?


PyDSTool.Toolbox.makeSloppyModel


| Home | Trees | Indices | Help | PyDSTool |
| --- | --- | --- | --- | --- |

Package PyDSTool ::
Package Toolbox ::
Module makeSloppyModel

[hide private]

[frames] | no frames]

# Source Code for Module PyDSTool.Toolbox.makeSloppyModel

```
  1  # makeSloppyModel: conversion from sloppy cell model description to PyDSTool
 
  2  # Robert Clewley, Oct 2005
 
  3  
 
  4  from PyDSTool import * 
  5  from copy import copy 
  6  
 
  7  allODEgens = findGenSubClasses('ODEsystem') 
  8  
 


9 -class sloppyModel(LeafComponent):


10      compatibleGens=allODEgens 
 11      targetLangs=targetLangs

 12  
 


13 -def makeSloppyModel(modelName, modelDict, targetGen, globalRefs=None,
 
 14                      algParams=None, silent=False):


15      if targetGen not in allODEgens: 
 16          print 'Valid target ODE solvers: ' + ", ".join(allODEgens) 
 17          raise ValueError('Invalid target ODE solver') 
 18      sModelSpec = sloppyModel(modelName) 
 19      if not silent: 
 20          print "Building sloppy model '%s'"%modelName 
 21  
 
 22      # first pass to collect names
 
 23      varnames = [] 
 24      for odeName in modelDict['odes'].keys(): 
 25          varnames.append(odeName) 
 26      parnames = [] 
 27      if 'parameters' in modelDict: 
 28          for parName in modelDict['parameters'].keys(): 
 29              parnames.append(parName) 
 30  
 
 31      pdomains = {} 
 32      xdomains = {} 
 33      if 'domains' in modelDict: 
 34          for name, dom in modelDict['domains'].iteritems(): 
 35              if name in parnames: 
 36                  pdomains[name] = dom 
 37              elif name in varnames: 
 38                  xdomains[name] = dom 
 39              else: 
 40                  raise ValueError("Name %s unknown in domain specs"%name) 
 41  
 
 42  
 
 43      for odeName, expr in modelDict['odes'].iteritems(): 
 44          if not silent: 
 45              print 'Adding ODE: ', odeName 
 46          if odeName in xdomains: 
 47              sModelSpec.add(Var(expr, odeName, specType='RHSfuncSpec',
 
 48                                 domain=xdomains[odeName])) 
 49          else: 
 50              sModelSpec.add(Var(expr, odeName, specType='RHSfuncSpec')) 
 51  
 
 52      auxvarnames = [] 
 53      if 'assignments' in modelDict: 
 54          for assgnName, expr in modelDict['assignments'].iteritems(): 
 55              if not silent: 
 56                  print 'Adding assignment: ', assgnName 
 57              sModelSpec.add(Var(expr, assgnName, specType='ExpFuncSpec')) 
 58              auxvarnames.append(assgnName) 
 59  
 
 60      if 'parameters' in modelDict: 
 61          for parName, val in modelDict['parameters'].iteritems(): 
 62              if not silent: 
 63                  print 'Adding parameter: ', parName, "=", val 
 64              if parName in pdomains: 
 65                  sModelSpec.add(Par(str(val), parName, domain=pdomains[parName])) 
 66              else: 
 67                  sModelSpec.add(Par(str(val), parName)) 
 68  
 
 69      auxfndict = {} 
 70      for funSig, expr in modelDict['functions'].iteritems(): 
 71          assert ')' == funSig[-1] 
 72          assert '(' in funSig 
 73          major = funSig.replace(')','').replace(' ','').split('(') 
 74          args = major[1].split(',') 
 75          name = major[0] 
 76          if not silent: 
 77              print 'Adding function: ', name, " of arguments:", args 
 78          sModelSpec.add(Fun(expr, args, name)) 
 79          auxfndict[name] = (args, expr) 
 80  
 
 81      if globalRefs is None: 
 82          globalRefs = [] 
 83      if not sModelSpec.isComplete(globalRefs): 
 84          print "Model retains free names: " + ", ".join(sModelSpec.freeSymbols) 
 85          print "These must be resolved in the specification before continuing." 
 86          print "If one of these is time, then include it explicitly as an" 
 87          print "entry in the argument list ('globalRefs' key)" 
 88          raise ValueError('Incomplete model specification') 
 89      targetlang = theGenSpecHelper(targetGen).lang 
 90      # single-generator model so give both same name
 
 91      if algParams is None: 
 92          algParams = {} 
 93      genName = modelName 
 94      sModel = ModelConstructor(modelName,
 
 95                              generatorspecs={genName: {'modelspec': sModelSpec,
 
 96                                                        'target': targetGen,
 
 97                                                        'algparams': algParams}}) 
 98      if not silent: 
 99          print "Adding events with default tolerances..." 
100      argDict={'precise': True, 'term': True} 
101      evcount = 0 
102      if 'events' in modelDict: 
103          for evspec, mappingDict in modelDict['events'].iteritems(): 
104              if evspec[:2] == 'lt': 
105                  dircode = -1 
106              elif evspec[:2] == 'gt': 
107                  dircode = 1 
108              else: 
109                  raise ValueError("Invalid event specification: use 'lt' and 'gt'") 
110              assert '(' == evspec[2], 'Invalid event specification' 
111              evspec_parts = evspec[3:].replace(')','').replace(' ','').split(',') 
112              evexpr = evspec_parts[0] 
113              threshval = evspec_parts[1] 
114              evname = 'Event'+str(evcount) 
115              argDict['name'] = evname 
116              ev = makeZeroCrossEvent(evexpr, dircode, argDict,
 
117                                         varnames, parnames, [], auxfndict, targetlang) 
118              evcount += 1 
119              sModel.addEvents(genName, ev) 
120              evmap = EvMapping(mappingDict, infodict={'vars': varnames+auxvarnames,
 
121                                                       'pars': parnames}) 
122              sModel.mapEvent(genName, evname, genName, evmap) 
123      if not silent: 
124          print "Building target model with default settings" 
125      return sModel.getModel()

126
```

  


| Home | Trees | Indices | Help | PyDSTool |
| --- | --- | --- | --- | --- |

|  |  |
| --- | --- |
| Generated by Epydoc 3.0.1 on Fri May 4 15:24:16 2012 | http://epydoc.sourceforge.net |
